# A 17 year old with isolated proximal tibiofibular joint arthritis

**DOI:** 10.1186/1546-0096-11-1

**Published:** 2013-01-09

**Authors:** Scott W Canna, Nancy A Chauvin, Jon M Burnham

**Affiliations:** 1 Division of Rheumatology The Children’s Hospital of Philadelphia, 3405 Civic Center Blvd, Philadelphia, PA, 19104, USA; 2 Department of Radiology The Children’s Hospital of Philadelphia, 3405 Civic Center Blvd, Philadelphia, PA, 19104, USA

**Keywords:** Ankylosing spondylitis, Proximal tibiofibular joint, Arthritis

## Abstract

The proximal tibiofibular joint (TFJ) is rarely affected in rheumatic diseases, and we frequently interpret pain of the lateral knee as the result of overuse or trauma. Nonetheless, the TFJ is a synovial joint that communicates with the tibiofemoral joint in a proportion of patients. While proximal TFJ arthritis has been rarely associated with existing spondyloarthritis, isolated TFJ arthritis as the presenting manifestation of spondyloarthritis has not yet been described. Here, we report the clinical and radiographic presentation of an adolescent with chronic proximal TFJ arthritis heralding spondyloarthritis highly suggestive of ankylosing spondylitis.

## Background

While pain and tenderness at the fibular head usually suggests trauma or overuse injury [1], arthritis should be included in the differential diagnosis. The proximal tibiofibular joint (TFJ) is a synovial-lined arthrodial plane joint, articulating between the lateral tibial condyle and the fibular head [2]. The joint is supported by the lateral collateral ligament, which attaches superiorly to the lateral femoral condyle. The TFJ mainly functions to dissipate rotational stress at the ankle, and provide tensile rather than weight-bearing support [2].

There is a single report of TFJ arthritis complicating existing Ankylosing Spondylitis (AS). *Hong et al.* reported evidence of TFJ arthritis in three of 16 known AS patients presenting for evaluation of knee pain [3]. All of these patients had concomitant radiographic findings consistent with AS. Here, we report a highly active adolescent male with isolated, chronic TFJ arthritis as the presenting sign of ankylosing spondylitis.

## Case presentation

**Figure 1:**
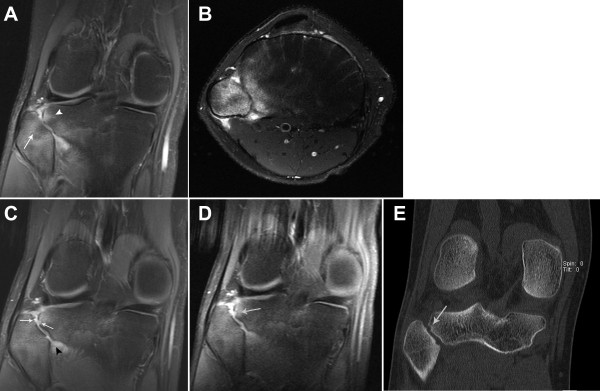
**Progression of right proximal tibiofibular joint (TFJ) arthritis.** (**A**) Coronal fat-saturated (FS) proton density MRI of the right knee shows bone marrow edema within the lateral aspect of the tibial plateau (arrowhead) and the fibular head (arrow). (**B**) Axial FS T2 sequence of the same joint demonstrating diffuse marrow edema of the fibular head and lateral tibial plateau. There is also adjacent soft tissue edema and a small amount of fluid within the proximal TFJ. (**C**) Follow-up coronal FS proton density sequence demonstrating an erosion at the site of prior bone marrow edema (arrows) as well as persistent inflammatory changes within the proximal TFJ (arrowhead). (**D**) Follow-up coronal FS T1 post gadolinium contrast-enhanced sequence again demonstrating the erosion seen in (**C**) (arrow) as well as enhancement of proximal TFJ synovium. (**E**) Reformatted coronal CT image of the same joint delineates the erosion at the lateral tibial plateau (arrow).

A 17-year-old Caucasian male presented to his orthopedist with a five-month history of lateral right knee and calf pain, with associated stiffness. There was no history of trauma, although he was an avid motorcross athlete. There was no family history of spondyloarthritis, chronic low back pain, or rheumatic diseases. Examination showed only tenderness over the right fibular head. Complete blood count, erythrocyte sedimentation rate, c-reactive protein concentration, antinuclear antibody, rheumatoid factor and Borrelia burgdorferi serologic testing were normal. A technetium bone scan demonstrated uptake of the lateral tibial condyle and fibular head. A subsequent noncontrast magnetic resonance imaging (MRI) showed only bony edema and soft tissue swelling of these same areas (Figures 1A & B). A Computed tomography (CT) scan performed 3 weeks later ruled out occult fracture and neoplasm.

He returned for orthopedic evaluation 8 weeks after the CT scan and an MRI with gadolinium showed a moderate TFJ effusion with persistent bony edema and surrounding synovial enhancement without synovial thickening. There was also a small subchondral erosion of the lateral tibia (Figure 1C & D). Upon review of his CT scan, the erosion was evident (Figure 1E).

**Figure 2:**
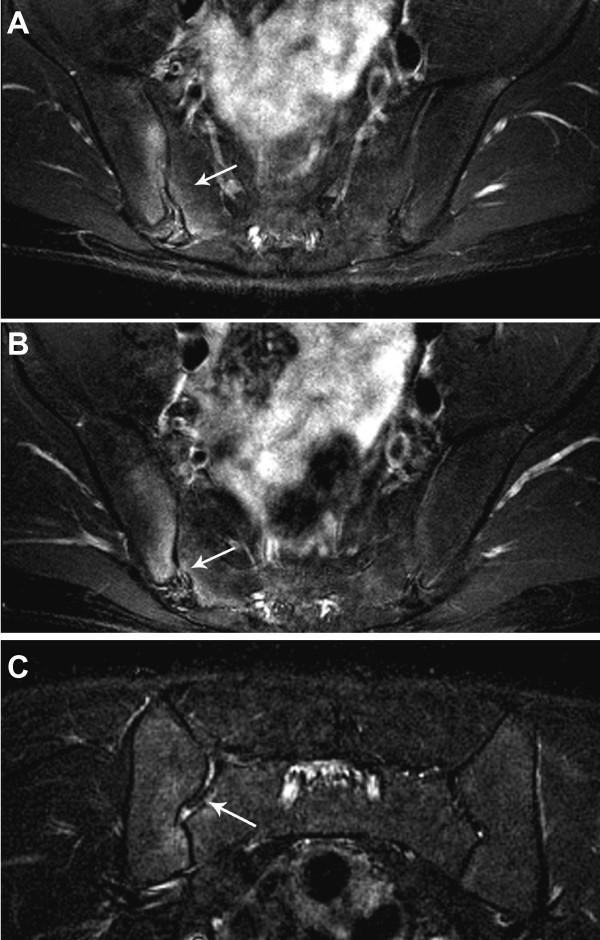
**Bone marrow edema indicative of early right sacroiliitis**. (**A**) Axial FS T2 sequence of the sacroiliac joints, at the level of the mid sacrum, shows edema of both the iliac and sacral (arrow) bones adjacent to the right sacroiliac joint. (**B**) Coronal FS T2 imaging demonstrates similar bone marrow edema as well as a small amount of fluid within the right sacroiliac joint (arrow).

Soon after his second MRI, and eight months after the onset of pain, he presented for rheumatologic evaluation. At this point he had been inactive in athletics for several months while his knee pain and stiffness gradually worsened. His examination was notable only for tenderness to the area of his right fibular head and an antalgic gait. His modified Schober measured 5.2 cm. Based on the unique location of the inflammatory process, he had a biopsy of the proximal tibia, which was unremarkable, but did not include any synovium. Anaerobic bacterial culture of the biopsy site grew a *corynebacterium* species after several days, and was felt to be a contaminant. Aerobic, fungal, and acid fast bacterial cultures were negative. In follow-up 8 weeks later, he had persistent lateral knee pain and now right sacroiliac pain and stiffness of 4 weeks duration, and his Schober was 4.9 cm. Radiographs done soon after the onset of sacroiliac pain were normal. Further evaluation revealed positivity for the Human Leukocyte Antigen (HLA)-B27 haplotype and an MRI of his pelvis revealed early right sacroiliitis (Figure 2). He began treatment with etanercept 50 mg weekly and showed dramatic improvement of his knee and sacroiliac pain and stiffness. His modified Schober had improved to 6.1 cm. He required a single intraarticular corticosteroid joint injection of the right knee for recurrence of stiffness and a small knee effusion 3 months after initiating etanercept. The knee arthritis subsequently resolved. After one year on etanercept, with occasional use of ibuprofen for lingering lateral right knee soreness, he remained stable with no new peripheral arthritis, uveitis, or further decline of lumbar mobility.

While this patient’s age at onset of symptoms exclude him from classification as enthesitis-related arthritis (ERA) or seronegative enthesopathy and arthropathy (SEA) syndrome, his presentation is relevant to the evaluation of younger patients [4,5]. He does not meet criteria for AS based on the modified New York criteria [6], but these criteria rely on X-ray evidence of sacroiliac involvement that may not be evident early in disease [7]. The Assessment of SpondyloArthritis International Society (ASAS) criteria are more appropriately applied to this patient. While he does not meet the criterion of > 3 months of inflammatory back pain, his axial imaging is strongly suggestive of sacroiliitis [8], and his HLA haplotype and peripheral arthritis strongly support an AS diagnosis [9].

## Discussion

Isolated inflammatory arthritis of the proximal TFJ joint should be included in the differential diagnosis of lateral knee pain and may require contrast-enhanced MRI to demonstrate synovitis. The most common cause for pain at this site is recurrent traumatic TFJ subluxation/dislocation, often seen with high-impact activities and which can result in bone marrow edema or other radiographic signs suggestive of inflammation [2,10]. The differential diagnosis of pain in this area should include infection, chronic arthritis (such as in rheumatoid arthritis), degenerative disease, neoplasm (e.g. osteosarcoma), pigmented villonodular synovitis (PVNS) and ganglion cyst [10,11]. In patients with a communication between the two joint spaces arthritis of one joint could, via transfer of inflammatory cells/mediators, result in arthritis of the other [10].

The relative rarity of TFJ arthritis should not overshadow its association with AS. TFJ arthritis masquerading as trauma or overuse could delay diagnosis or treatment of axial disease in AS. Increased time between symptom onset, diagnosis, and treatment have been associated with more severe clinical and radiographic disease in AS [12]. Thus, when a practitioner confirms proximal TFJ arthritis, *consideration* for occult signs of spondyloarthritis should follow.

## Conclusions

Inflammatory arthritis of the proximal tibiofibular joint is an uncommon but likely underdiagnosed cause of lateral knee pain. Arthritis of this joint has been reported in connection with ankylosing spondylitis. Practitioners should be careful to evaluate for arthritis as part of a work-up for chronic lateral knee pain and stiffness, and should consider concomitant evaluation for spondyloarthritis.

## Consent

Written informed consent was obtained from the patient for publication of this Case Report and any accompanying images. A copy of the written consent is available for review by the Editor-in-Chief of this journal.

## Competing interests

The authors declare that they have no competing interests

## Authors’ contributions

SC collected the clinical information and wrote the case report. NC and JB critically reviewed and edited the manuscript. All authors read and approved the final manuscript.
